# Give me a break! Unavoidable fatigue effects in cognitive pupillometry

**DOI:** 10.1111/psyp.14256

**Published:** 2023-02-03

**Authors:** Drew J. McLaughlin, Maggie E. Zink, Lauren Gaunt, Jamie Reilly, Mitchell S. Sommers, Kristin J. Van Engen, Jonathan E. Peelle

**Affiliations:** 1 Department of Psychological and Brain Sciences, Washington University in Saint Louis, St. Louis, Missouri, USA; 2 Department of Otolaryngology, Washington University in Saint Louis, St. Louis, Missouri, USA; 3 Department of Communication Sciences and Disorders, Temple University, Philadelphia, Pennsylvania, USA

**Keywords:** experiment design, fatigue, growth curve analysis, pupillometry

## Abstract

Pupillometry has a rich history in the study of perception and cognition. One perennial challenge is that the magnitude of the task-evoked pupil response diminishes over the course of an experiment, a phenomenon we refer to as a fatigue effect. Reducing fatigue effects may improve sensitivity to task effects and reduce the likelihood of confounds due to systematic physiological changes over time. In this paper, we investigated the degree to which fatigue effects could be ameliorated by experimenter intervention. In Experiment 1, we assigned participants to one of three groups—no breaks, kinetic breaks (playing with toys, but no social interaction), or chatting with a research assistant—and compared the pupil response across conditions. In Experiment 2, we additionally tested the effect of researcher observation. Only breaks including social interaction significantly reduced the fatigue of the pupil response across trials. However, in all conditions we found robust evidence for fatigue effects: that is, regardless of protocol, the task-evoked pupil response was substantially diminished (at least 60%) over the duration of the experiment. We account for the variance of fatigue effects in our pupillometry data using multiple common statistical modeling approaches (e.g., linear mixed-effects models of peak, mean, and baseline pupil diameters, as well as growth curve models of time-course data). We conclude that pupil attenuation is a predictable phenomenon that should be accommodated in our experimental designs and statistical models.

## 1 | INTRODUCTION

Pupillometry has a long history of use for indexing autonomic and cognitive effects across a wide range of tasks (Kahneman & Beatty, 1966; Laeng et al., 2012; for a review of use in speech perception, see Van Engen & McLaughlin, 2018). By examining the change in the size of the pupil following presentation of a cognitively demanding stimulus (“cognitive” pupillometry), one can compare the relative difficulties of multiple experimental conditions. One challenge for cognitive pupillometry is that the pupil response may also change with non-experimental factors (Tryon, 1975). For example, over the course of a 30-min experiment, pupil responses will progressively diminish in amplitude even when task demands remain constant (Brown et al., 2020). For consistency, we will refer to these amplitude reductions as a “fatigue effect”; however, it may also reflect habituation to the task or stimuli (Morad et al., 2000; Tryon, 1975).

Research in monkeys and humans indicates that pupillary dynamics covary with activity in the locus coeruleus (Aston-Jones & Cohen, 2005; Gilzenrat et al., 2003; Rajkowski, 1993), a neuromodulatory nucleus in the brainstem that is particularly important for attention-demanding tasks (Berridge & Waterhouse, 2003; Samuels & Szabadi, 2008). Neurons in the locus coeruleus have two types of activity: tonic (baseline activity) and phasic (stimulus-driven activity). In the context of cognitive pupillometry, researchers typically focus on phasic activity, or, in other words, the *task-evoked* pupil response (Beatty, 1982). However, a large amount of research on the locus coeruleus system indicates that tonic and phasic activity interact (Aston-Jones & Cohen, 2005; Berridge & Waterhouse, 2003). Pupil responses depend on an interaction between tonic activity (which may reflect arousal and/or attention) and phasic activity (which, in a typical cognitive experiment, primarily reflects the response to an experimental stimulus). When tonic activity is low, phasic activity will also be low (and behavioral performance typically poorer); when tonic activity is in an intermediate range, phasic activity, and behavioral performance will be optimal; and when tonic activity is especially high (i.e., due to stress), phasic activity will be low (and behavioral performance typically poorer).

For cognitive pupillometry, researchers sometimes report that the task-evoked pupil response (a phasic response) shrinks in amplitude over the course of an experiment (Hopstaken et al., 2015; McGarrigle et al., 2017; Pielage et al., 2021). This change in phasic activity may be linked to a change in tonic activity: If participants begin the task at an intermediate level of tonic activity, and slowly become disengaged with the task (due to fatigue, habituation, or boredom), then tonic activity will steadily drop, and phasic responsiveness of the pupil along with it.

Fatigue effects are a major concern for several reasons. First, broadly speaking, we hypothesize that a reduced response may affect the optimal statistical model and our sensitivity to examine experimental manipulations. Second, for experimental paradigms where adaptation or habituation is of scientific interest, disentangling stimulus-specific effects from global pupil fatigue is crucial (e.g., Brown et al., 2020). A question for pupillometry researchers, then, is whether fatigue effects might be reduced through specific experimental protocols.

Although we refer to decreases in pupil amplitude as a “fatigue” effect, we note that the construct of fatigue is complex, encompassing both subjective feelings of weariness, lack of energy, or decreased motivation as well as physical decrements (Tiesinga et al., 1996; Chaudhuri & Behan, 2000; see also comprehensive review by Hornsby et al., 2016). For cognitive pupillometry, it remains unclear how to best characterize and mitigate fatigue of the pupil response. Some work by Hopstaken et al. (2015) has indicated that task engagement may be a key factor. The authors examined peak pupil diameter across a 2 h *n*-back memory task and found steady decrease across blocks of the task. However, in a final block, they added a reward incentive (an offer to shorten the length of the block if the subject showed improved performance) and found a large increase in the size of the peak pupil diameter. This change following the incentive block provides positive evidence that, with sufficient motivation, increased task engagement may restore the amplitude of the pupil response. What remains to be determined is whether fatigue of the pupil response can be steadily prevented and reduced across an experimental task via task engagement, and whether less extreme experimental protocols (such as breaks from the task) may provide a similar benefit.

One suggestion (mentioned by Winn et al., 2018) is that frequently engaging with a participant during an experiment may reduce fatigue effects. However, there is a paucity of empirical evidence regarding how effective this approach is, or whether there might be additional ways to optimize an experimental protocol. For example, when a researcher interacts with a participant, several things happen:

1. The participant stops performing the experimental task (i.e., they get a break);
2. The participant engages in a social exchange;
3. The participant engages in a task other than the experimental task;
4. The participant is reminded that the researcher is monitoring them.

It could be that, independently, one of these manipulations is particularly effective at reducing fatigue effects.

Indeed, work examining participants with hearing loss has shown that heart rate (a correlate of pupil diameter; Wang et al., 2018) can be affected by experimenter observation, possibly reflecting the physiological stress caused by social evaluation (Plain et al., 2021). Examining the task-evoked pupil response directly, Pielage et al. (2021) found that, for young adults with normal hearing, when participants completed the task with another participant in the same room they had larger peak pupil responses overall as compared to when they completed the task in a room alone. What remains to be examined, however, is the direct effect of observation on the fatigue of the pupil response across an experiment.

As a proof of concept that we could influence fatigue effects, we conducted a pilot study (details reported in Supplemental Materials). Twelve participants (six per group) were recruited and assigned to one of two groups: control (i.e., no breaks or observation), and intervention (i.e., breaks involving social interaction and observation). As shown in Supplemental Figure S1, we observed what appeared to be a marked reduction in fatigue effects (i.e., less change in the magnitude of the pupil response over trials) when a researcher was present and administered “social breaks,” compared to when participants paced themselves through the task with no breaks and no one else in the room. Encouraged by these pilot data, we set out to systematically evaluate the degree to which we could reduce fatigue effects and better isolate the factors contributing to any reduction. We implemented the following task manipulations: whether breaks were offered to the subject during the experiment, the degree of social interaction with the research assistant, and whether participants were (knowingly) monitored by the research assistant.

Specifically, in Experiment 1 we included the following between-subject conditions: control (no breaks), kinetic breaks (breaks provided, non-social activity), and social breaks (breaks provided, social activity). During breaks, participants assigned to the kinetic breaks condition interacted with a highly tactile toy of their choice (details in Method), whereas participants assigned to social breaks condition engaged in casual conversation with the research assistant. We structured the social breaks condition in accord with recommendations on best practices for optimizing data fidelity in pupillometry (e.g., Winn et al., 2018) and as guided by our own pilot data. The kinetic breaks condition served as an active control involving nominal physical activity devoid of secondary linguistic and social demands. For Experiment 1, we predicted that both the kinetic and social interventions would reduce pupillary fatigue relative to the control condition.

In Experiment 2, we examined the effects of researcher observation of the participants in conjunction with the effect of social breaks, resulting in the following conditions: control (no breaks, no observation), observation (no breaks, observation), and observation with breaks (social breaks, observation). This manipulation was designed to assess the impact of the “good subject effect” on response behavior, a robust phenomenon linking task performance in laboratory settings with a desire to satisfy the experimenters (Nichols & Maner, 2008). We predicted that participants who were knowingly observed by a research assistant would show less fatigue, and that this manipulation of direct supervision combined with social breaks would be the most effective intervention.

Additionally, in both experiments, we explored change in baseline pupil diameter across the task (indexing tonic arousal). Prior work has demonstrated mixed outcomes when examining changes in baseline pupil diameter across a task; Pielage et al. (2021) found that baseline pupil diameter consistently decreased across task blocks regardless of the ordering of their primary manipulation (observation, discussed above), whereas Hopstaken et al. (2015) found no significant changes in baseline pupil diameter across task blocks. We anticipated that examining trends in baseline pupil diameter in conjunction with the task-evoked (phasic) pupil response would prove informative, given these conflicting outcomes. Our prediction was that baseline pupil size would decrease across the task for all participants, but that less decrease in baseline pupil diameter would be present for participants assigned to one of our intervention conditions as compared to our control conditions.

Finally, we incorporate multiple analysis methods common in cognitive pupillometry for the present study. In our analyses of both experiments, we analyze the mean pupil diameter, peak pupil diameter, baseline pupil diameter, and the full time-course of the data (using growth curve analysis; Mirman, 2017). These varying approaches allowed us to compare how the sensitivity of these different analysis tools may be modulated by fatigue effects. Growth curve analysis, for example, allows the researcher to model change over time in the size of the pupil in response to a stimulus, and incorporate individual differences in pupil response functions across subjects (see Hershman et al., 2022). In contrast, measures of mean, peak, and baseline pupil diameter summarize the complex pupil response functions into their most informative features. If these approaches to data management and modeling are differentially sensitive to the fatigue of the pupil response (i.e., effects of interest are more or less obscured by the large effect of pupil response fatigue), this will be crucial information for researchers analyzing pupillometry data.

## 2 | EXPERIMENT 1

### 2.1 | Method

#### 2.1.1 | Data and code availability statement

Materials, data, analysis scripts, and pre-registration are available from: https://osf.io/jkcx5/.

#### 2.1.2 | Participants

Participants (*n* = 96) were recruited using the Washington University in St. Louis Psychology Human Participants Pool. Seven participants were excluded due to data loss from blinking, and data for two additional participants were lost due to technical error, resulting in 87 participants with valid data (29 control, 28 kinetic breaks, 30 social breaks).

Due to COVID-19 safety regulations, data collection was prematurely halted before the pre-registered target sample size (90 useable participants) was reached. The age range of the participants was 18–29 years (M = 19.8, SD = 1.5). Participants provided informed consent and received course credit or pay (at a $10/h rate) as compensation for participation. All procedures were approved by the Washington University Institutional Review Board. Participants were native speakers of English with self-reported normal hearing. Of the 87 participants included in the sample, 57 self-identified as female and 30 self-identified as male.

#### 2.1.3 | Materials

##### Speech stimuli

A female, American-English native speaker was recorded reading sentences developed by Van Engen et al. (2012), containing four keywords each (“the *hot sun warmed* the *ground*”). Speech-shaped noise was created from these stimuli using Praat version 6.0.16 (Boersma & Weenink, 2019), and then mixed with the targets at −5 dB SNR using *jp_addnoise.m* from http://github.com/jpeelle/jp_matlab. Noise began three seconds before sentence onset and followed for three additional seconds after sentence offset. These sentences have previously been shown to result in robust task-evoked pupil responses even when presented in quiet (McLaughlin & Van Engen, 2020).

##### Kinetic activities

**FIGURE 1:**
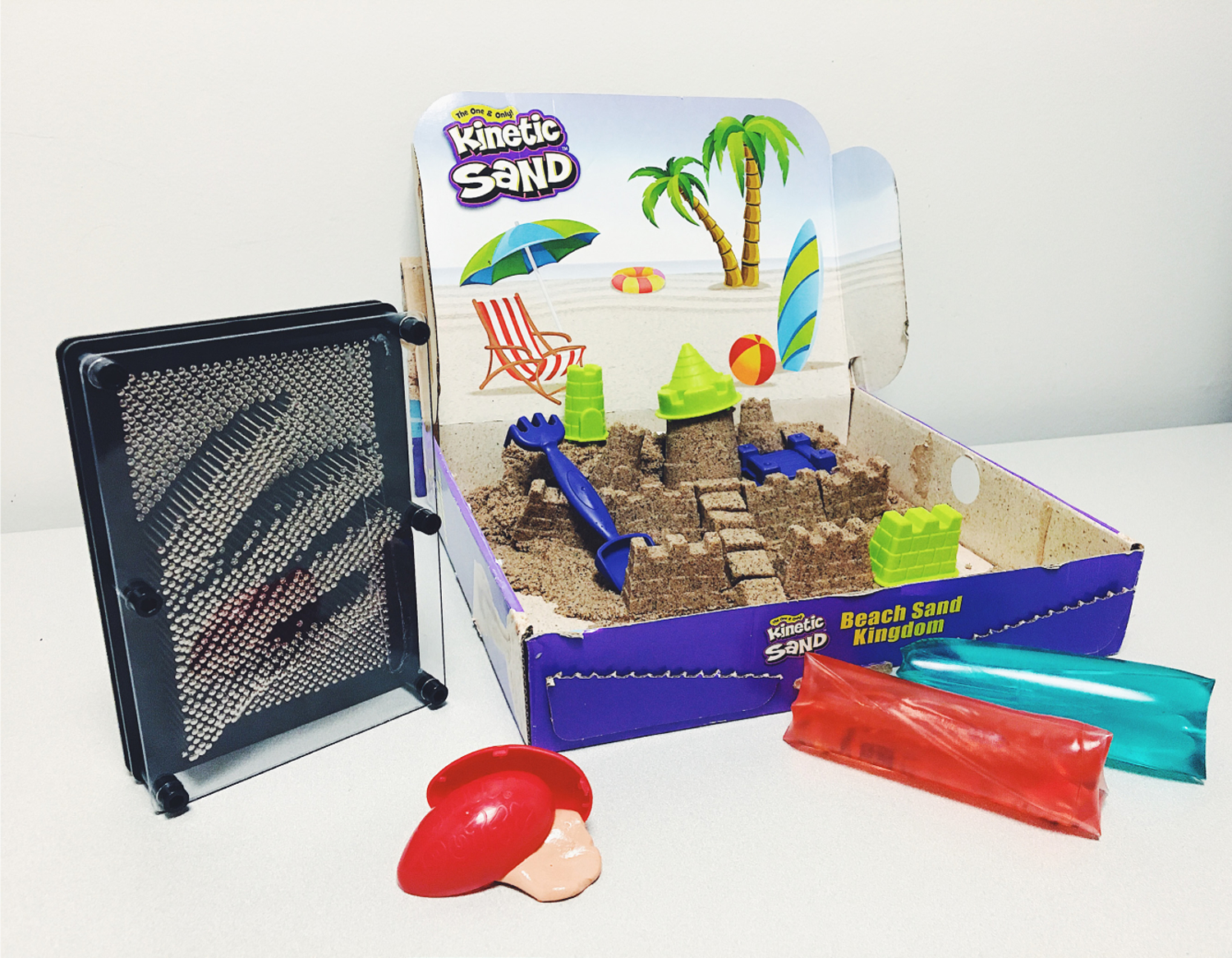
Picture of the four toys offered to participants in the Kinetic Breaks condition.

Participants assigned to the Kinetic Breaks condition were presented with the choice of four kinetic activities during breaks (Figure 1). The activities included: (1) Silly Putty, a bouncing, silicone-based putty, (2) a Kinetic Sand Beach Kingdom Playset with 3 pounds of beach sand, (3) 5-inch water wigglers with colorful beads and glitters, and (4) a 5 × 7 inch Pinart 3-Dimensional Pin Sculpture Board. Participants were permitted to switch between the activities freely.

### 2.2 | Procedure

Participants were randomly assigned to one of three conditions: a Control condition, in which participants completed all trials with no breaks; a Kinetic Breaks condition, in which participants engaged with their choice from an array of kinetic toys and activities during breaks; and a Social Breaks condition, in which the researcher engaged the participant in conversation during breaks.

All participants provided written informed consent prior to study participation. Participants were seated in a sound-attenuating booth facing a monitor and EyeLink 1000 camera. All equipment was set at distances following EyeLink specifications. Participants rested their chins on a head mount during the task. The EyeLink camera was calibrated to eye movement using a nine-point calibration and validation. The task pupil diameter was recorded at 500 Hz from the left eye for all participants.

The experiment began with a practice session lasting five trials. Next, participants completed 80 test trials, during which participants assigned to break conditions received seven breaks (interspersed every 10 trials). In both break conditions, participants were escorted to an adjacent room to complete their assigned break activity. Breaks were timed to last 60 s (beginning when the participant entered the break room). Calibration of the EyeLink camera to the pupil was visually inspected by the research assistant before the participant resumed the experiment after each break. Participants in the Control condition did not receive breaks during the task. The total duration of the experiment was approximately 45 min for the Control group, and 55 min for the Social Breaks and Kinetic Breaks groups.

**FIGURE 2:**
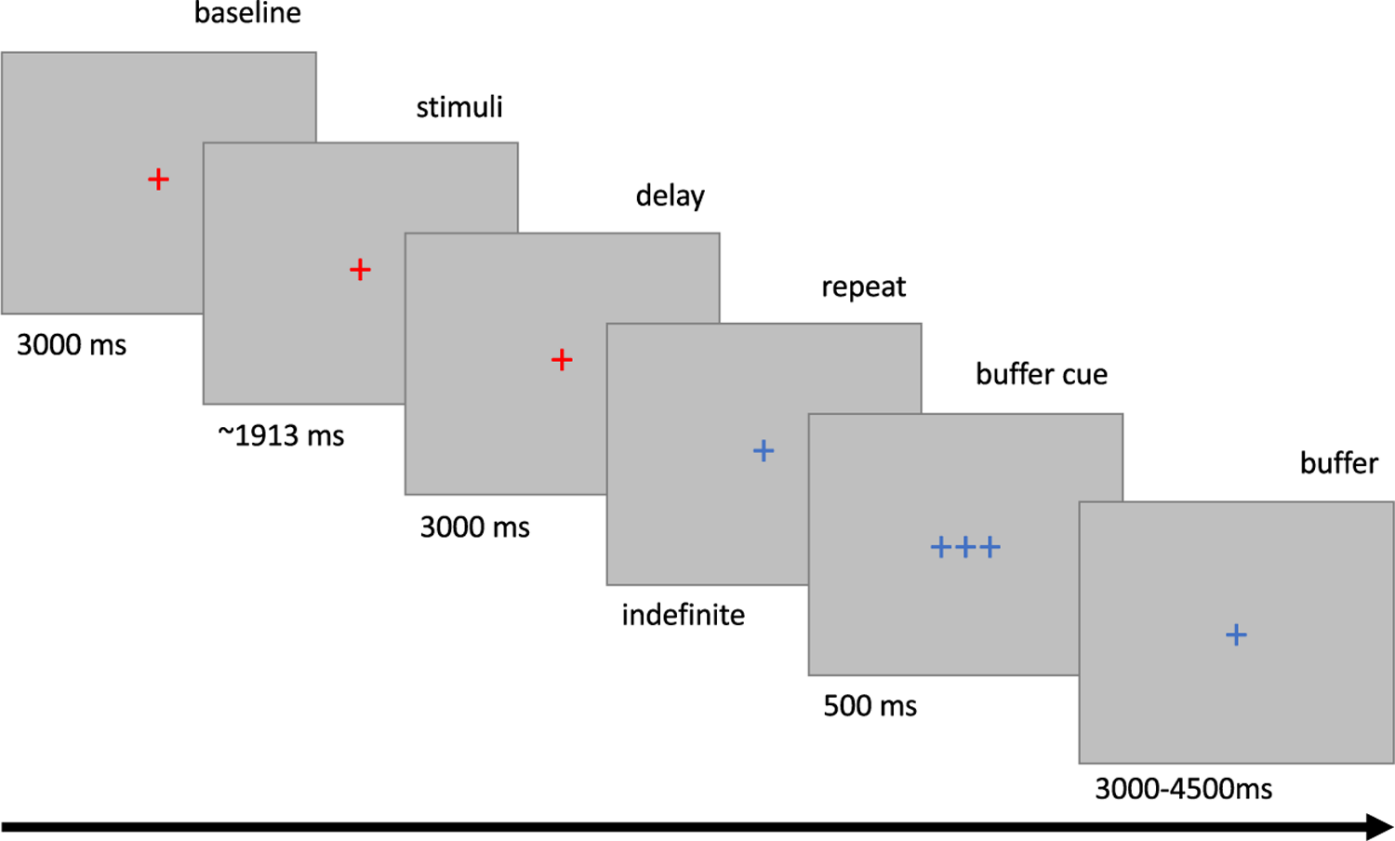
Timings for the pupillometry task. All data collection occurred during sections with red fixations crosses.

The trial flow is depicted in Figure 2. Participants were instructed to fixate on a cross located in the center of the screen at all times. Pupil diameter was recorded during all periods in which the color of the cross was red, and participants were asked to reduce their blinking (to a comfortable degree) during these times. All groups were presented with the same 80 speech-in-noise files, presented in a random order. Each stimulus was preceded by a 3000 ms baseline period of speech-shaped noise (used during analysis to establish pupil size prior to the target sentence) and followed by a 3000 ms delay period, during which the speech-shaped noise continued. Thus, background noise was present throughout the recording window. After this window, the color of the cross changed to blue, cueing the participant to repeat what they heard aloud for the recording device. Participants then used a Chronos foot pedal to continue to the next trial. After pressing the foot pedal, a brief cue (three fixation crosses) flashed on the screen for 250 ms to confirm that the next trial was loading. Here, a randomized interstimulus interval (ISI) was inserted to allow additional time for the pupil response to recover between trials. To reduce the predictability of experiment timing, the ISI varied, lasting 3000, 3500, 4000, or 4500 ms. All participants completed a demographic, language, and motivation questionnaire and were debriefed after concluding the experiment. All procedures were approved by the Washington University in St. Louis Institutional Review Board.

#### 2.2.1 | Data preparation and analysis

Pupillometry data were prepared for analyses in R version 4.0.4 (R Core Team, 2013, RRID:SCR_001905) using functions from the *gazeR* package (Geller et al., 2020). First, trials missing more than 50% of timepoints were removed, and participants missing more than 20% of trials were excluded. Next, blinks (periods of missing data) were identified, extended 100 ms prior and 200 ms following, and interpolated across linearly. A five-point moving average was applied to smooth the data. Baseline pupil size was then calculated and subtracted from all timepoints for every trial within each subject (Reilly et al., 2019). The window of data used to calculate the baseline value of each trial was the 500 ms immediately preceding the onset of the target sentence. The final pre-processing step involved downsampling the pupil data, such that sampling frequency was reduced from 500 Hz to 50 Hz. Downsampling the data substantially reduces computational demand and can also reduce temporal autocorrelation in the data (van Rij et al., 2019).

For peak and mean pupil analyses, the pre-processed data were then summarized by subject and trial using functions from the *dplyr* package (Wickham et al., 2021). For peak and mean pupil diameter, data were first trimmed to include only data from 300 ms (approximately the earliest possible onset of the pupil response) to 2500 ms (approximately 500 ms after the peak of the pupil response) before calculating average values. Our pre-registration stated that our analysis window would begin at 0 ms, but we opted to change this for both the peak and mean pupil diameter analyses, as well as the growth curve analyses (GCA), to 300 ms before any of the results were known. The starting point of 300 ms was selected due to the delay in the pupil response, which is often found for cognitive pupillometry. For GCA, the starting point of 300 ms was also better suited to a polynomial fit than the starting point of 0 ms. For peak and mean pupil diameter, limiting the window of data for analyses can also reduce the likelihood of spurious high values (e.g., values recorded in error by the eye tracker that may not have been caught during pre-processing).

This same analysis window was also selected for the GCA of the data. Our aim in selecting this window was to include as much of the pupil response curve as possible within the constraints of a typical polynomial shape (cubic, in this case). A plot of the data with a base growth curve model was created with only the polynomial terms (linear, quadratic, and cubic) and random intercepts by subject were used to confirm that a time window of 300 ms to 2500 ms (where 0 ms is the start of the sentence) would be well-suited for the shape of the pupil response. The raw data were collapsed across all conditions to prevent researcher bias. Visual inspection was used to determine that the model-predicted fit line approximated the raw data sufficiently. To avoid increasing researcher degrees of freedom (Peelle & Van Engen, 2021), all decisions about the analysis window were made prior to analyzing the fixed effects of interest.

### 2.3 | Results

#### 2.3.1 | Peak and mean pupil diameter

**FIGURE 3:**
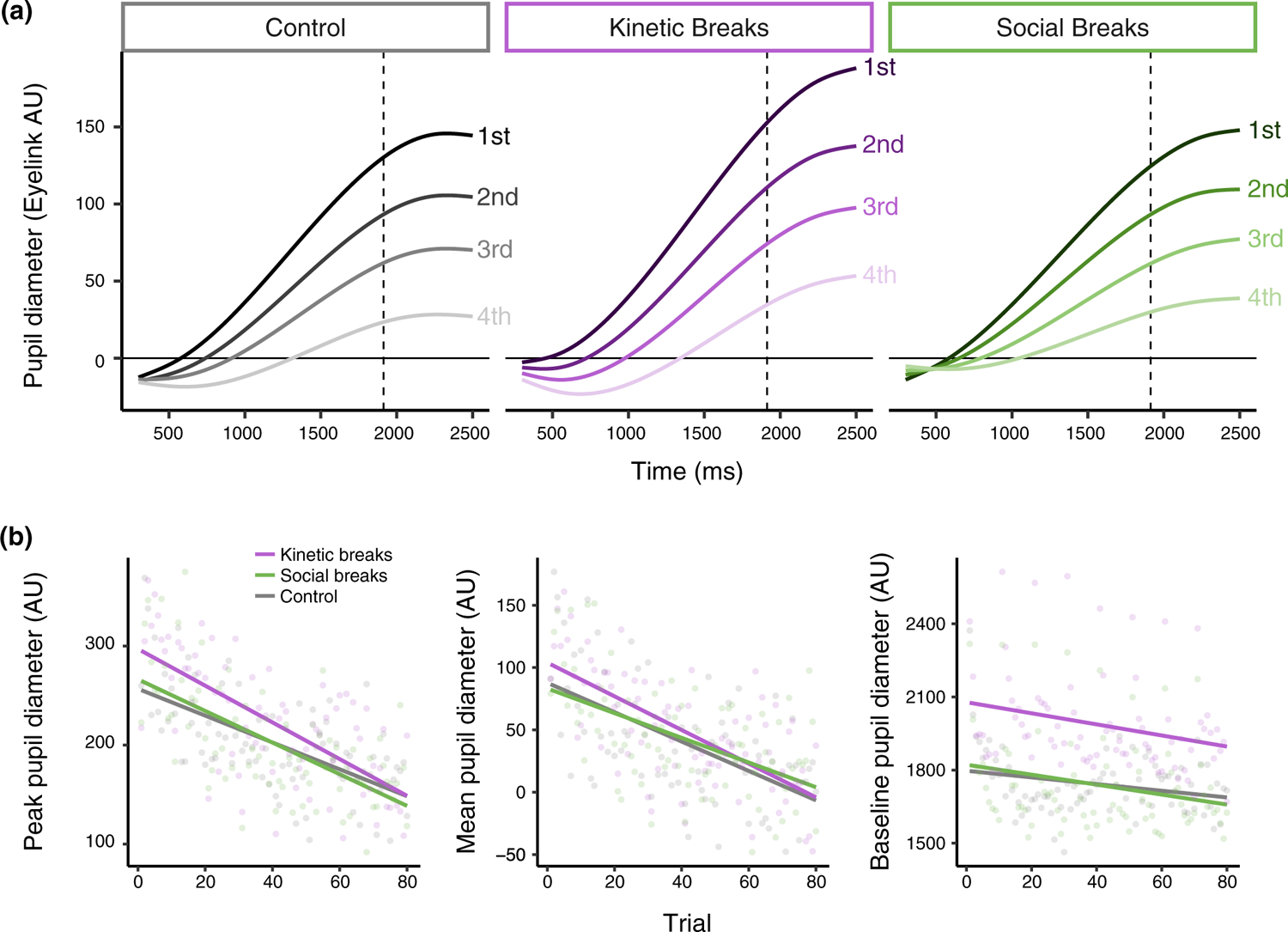
(a) Fatigue of the pupil response in Experiment 1 is visualized by summarizing predicted GCA fits by quartile (i.e., collapsing trials 1–20, 21–40, 41–60, and 61–80). Quartiles of trials (“1st,” “2nd,” “3rd,” and “4th” lines) are for visualization only and were not used for analyses. Size of the pupil is shown on the y-axis, and time within the trial (where zero is the start of the stimulus) is shown on the x-axis. Dashed vertical lines show the average offset of the stimuli. (b) Changes in peak pupil diameter (left), mean pupil diameter (middle), and baseline pupil diameter (right) across trials are shown for each random assignment group of Experiment 1. Lines represent model fits and points represent mean values of the raw data summarized across all participants for each trial. Note that the peak and mean pupil diameter measures reflect baselined values, whereas baseline pupil diameter is a summary of the raw arbitrary units used for the baselining process.

Linear mixed-effects regression and log-likelihood model comparisons were used to examine the peak and mean pupil diameter. Random effects included intercepts by subject and by item (i.e., sentence). Models were unable to converge when random slopes of trial by subject were included. Fixed effects included group (levels: control, kinetic breaks, social breaks), trial, and the interaction between group and trial. For peak pupil response, the effect of group did not significantly improve model fit (*χ*^2^(2) = 0.74, *p* = .69). The effect of trial, however, made a large improvement to model fit (*χ*^2^(1) = 218.17, *p* < .001), with the direction of the effect indicating that peak pupil size reduced over the course of the experiment, as expected (*β* = −1.59, SE = 0.11, *t* = −14.89, *p* < .001). Lastly, the interaction between group and trial did not improve model fit significantly (*χ*^2^(2) = 3.28, *p* = .19), although model estimates did indicate a marginal difference between the control and kinematic groups (*β* = −0.48, SE = 0.26, *t* = −1.81, *p* = .07). As shown in Figure 3b, the rate of decrease in peak pupil response across the experiment was somewhat steeper for the kinetic breaks group than the control group. The direction of this interaction is in the opposite direction of what we predicted and would indicate that participants given no breaks (the control group) actually showed less fatigue of the pupil response over the task.

For mean pupil response, the effect of group once again did not significantly improve model fit (*χ*^2^(2) = 0.29, *p* = .86), while the effect of trial did (*χ*^2^(1) = 138.35, *p* < .001). The model estimate for trial indicated that the magnitude of the pupil response decreased across the course of the task (*β* = −1.16, SE = 0.10, *t* = −11.82, *p* < .001). Unlike in the analysis of the peak pupil response, however, in the model of mean pupil response the interaction between group and trial did not improve model fit (*χ*^2^(2) = 2.11, *p* = .35; Figure 3b).

#### 2.3.2 | Growth curve analysis

For the growth curve analysis (GCA) of the time-course of the pupil response, the random effect structure included random intercepts by subject and item, and random slopes of each polynomial by subject and item. The specifications for the random slopes by item were slightly simplified by removing correlations among the random effects. In two cases, reduced models (e.g., a model without the interaction effect of Group and Trial, made to compare against a full model which includes the effect) were unable to converge and the random effect structure was simplified further by removing random slopes for the cubic polynomial by item. Under ideal circumstances, a full random effect structure would be implemented, but was not possible in the case of this data set. Using a fuller random effect structure for GCA can help to reduce the potential of autocorrelation in the data, and inflated Type 1 error rate (see Jiang et al., 2017).

For fixed effects, we tested the contributions of each polynomial, group, trial, and interactions among each of these factors. Log-likelihood model comparisons were used to determine whether each effect significantly improved model fit (summarized in Table 1), and model summaries were used to examine the direction of effects (summarized in Appendix A).

The linear (*χ*^2^(1) = 59.16, *p* < .001) and cubic (*χ*^2^(1) = 30.88, *p* < .001) polynomials improved model fit, but the quadratic polynomial did not (*χ*^2^(1) = 0.01, *p* = .91). However, because subsequent interactions with trial were significant (noted below), we decided to include up through the cubic polynomial. The effect of group did not significantly improve model fit (*χ*^2^(2) = 1.15, *p* = .56), but the effect of trial did (*χ*^2^(1) = 11,705, *p* < .001).

Next, we tested whether each of the model interactions improved fit significantly. The interaction between group and trial was significant (*χ*^2^(2) = 158.90, *p* < .001; Figure 3a). Model estimates indicated that, compared to the control group, pupil response for the kinetic breaks group reduced more rapidly over trials (*β* = −0.15, SE = 0.03, *t* = −5.79, *p* < .001), and pupil response for the social breaks group reduced less rapidly over trials (*β* = 0.18, SE = 0.03, *t* = 6.81, *p* < .001). In other words, there was less fatigue of the pupil response for participants who received social breaks. However, as visible in Figure 3a, while significant differences between conditions emerged, the decrease in pupil response across trials was still very large in all of the conditions. Additionally, the kinetic breaks group’s pupil response started at a higher value than the other conditions. Supplemental Figure S2 shows the non-linear change across trials using raw data (in place of model fits).

The interactions between the effect of group and the linear (*χ*^2^(2) = 1.20, *p* = .55), quadratic (*χ*^2^(2) = 2.91, *p* = .23), and cubic (*χ*^2^(2) = 1.75, *p* = .42) polynomials were all non-significant. Interactions with the effect of trial, however, were all significant (*χ*^2^(1) = 3525.60, *p* < .001; *χ*^2^(1) = 58.38, *p* < .001; *χ*^2^(1) = 19.85, *p* < .001). The model estimates indicated that, across trials, the pupil response curves became shallower (interaction of linear polynomial and trial: *β* = −6.66, SE = 0.11, *t* = −59.45, *p* < .001), and the inflections of the pupil response curves became flatter (interaction of quadratic polynomial and trial: *β* = 0.86, SE = 0.11, *t* = 7.64, *p* < .001; interaction of cubic polynomial and trial: *β* = 0.50, SE = 0.11, *t* = 4.46, *p* < .001).

**FIGURE 4:**
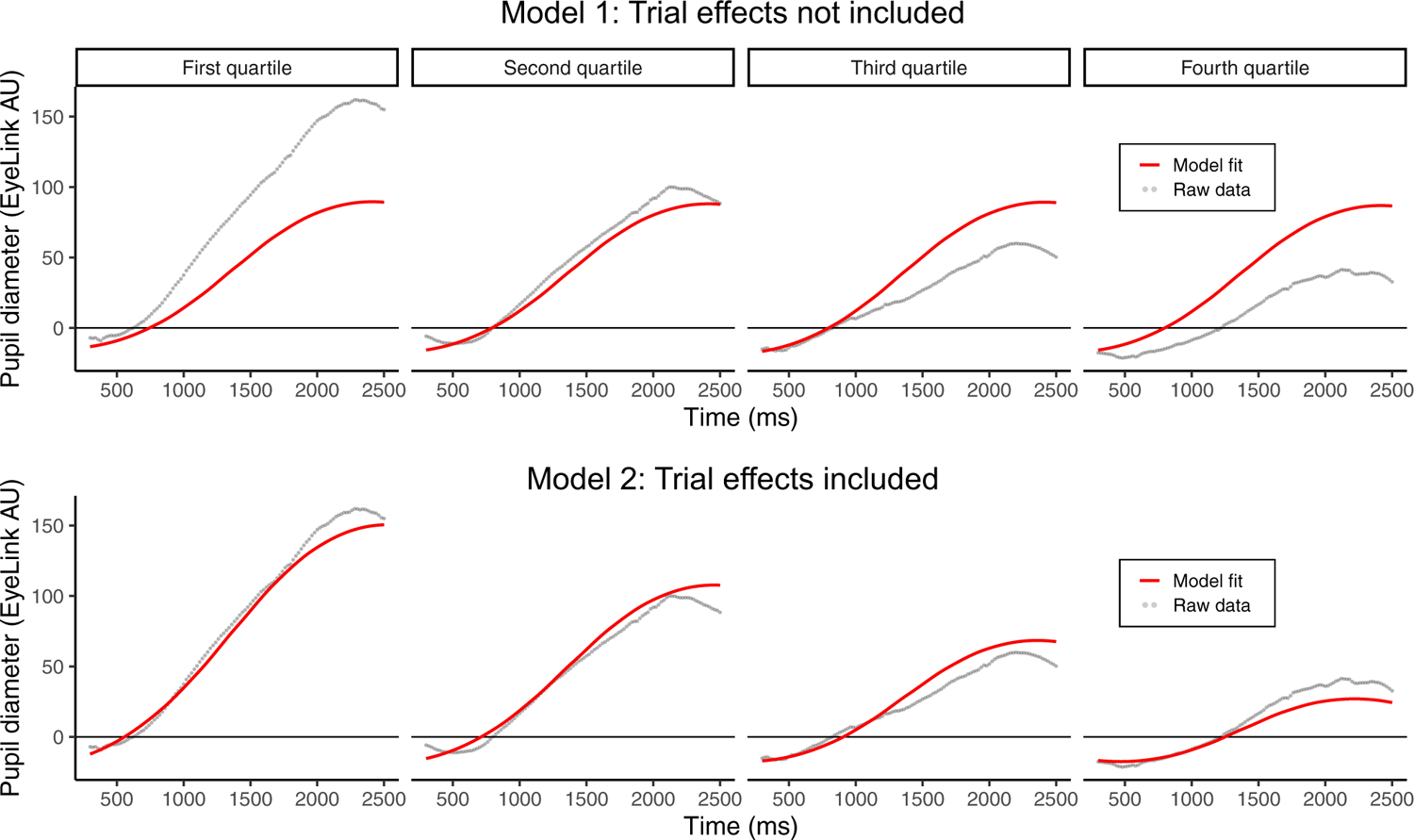
Visualization of the importance of modeling trial (i.e., time across the experiment) is shown using data from the control condition in Experiment 1. Here, raw data (gray dots) and predicted GCA fit lines are summarized by quartile (i.e., collapsing trials 1–20, 21–40, 41–60, and 61–80). Quartiles of trials are for visualization only and were not used for modeling. Size of the pupil is shown on the y-axis, and time within the trial (where zero is the start of the stimulus) is shown on the x-axis.

Here, we also note the large effect of the trial predictor. As part of our model comparisons (conducted in *R* with the *anova()* function), we also calculated Bayesian Information Criterions (BICs). By comparing the BIC of our reduced model without the trial effect (*BIC* = 10,237,675) against the BIC of our model with the trial effect (*BIC* = 10,225,983), we were able to estimate the difference in BIC to be: *ΔBIC* = 11,692 (favoring the model with the effect of trial; Raftery, 1995). Given that a *ΔBIC* score greater than 10 is typically interpreted as “very strong evidence,” it is safe to conclude that the size of the trial effect in the present study is extremely large, and dramatically improves model fit. Examination of the chi-square estimates in Table 1 also demonstrates that the size of the trial effect is dramatically larger than the other effects—including even the polynomial terms. Further, the model estimate for trial (*β* = −1.16, SE = 0.01, t = −108.61, *p* < .001) indicates a decrease of 1.16 EyeLink Arbitrary Units (AU) each trial. To put this value into perspective, we used the model-predicted values from the growth curve analysis to estimate a percent decrease in the height of the pupil curve (i.e., peak pupil diameter) across the experiment. Using these estimates, we determined that the peak on Trial 1 is approximately 139 AU, while the peak on Trial 80 is approximately 55 AU; thus, the peak pupil response is estimated to have decreased 60% across the 80-trial experiment. Lastly, we visualize the importance of including the effect of trial (as well as interactions between trial and the orthogonalized polynomials) in Figure 4 using data from the control condition of Experiment 1.

#### 2.3.3 | Exploratory analyses of baseline pupil diameter

One subject in the control condition was identified as having impossibly low baseline values (more than 43 standard deviations below the mean), likely due to computer error, and was subsequently excluded from the analyses. Including versus excluding their data did not change the conclusions of the analyses of the baseline pupil diameter.

We used linear mixed-effects regression to examine baseline pupil diameter. Fixed effects included trial and group, and the interaction between trial and group. Random effects included random intercepts by subject and item. Log-likelihood model comparisons indicated that trial significantly improved model fit (*χ*^2^(1) = 86.68, *p* < .001), and the model estimate indicated a decrease in baseline pupil diameter across trials (*β* = −1.82, SE = 0.19, *t* = −9.34, *p* < .001). The effect of group did not significantly improve model fit (*χ*^2^(2) = 2.36, *p* = .31), but the interaction between trial and group did (*χ*^2^(2) = 6.61, *p* = .04). The model estimates indicated that baseline pupil diameter decreased more rapidly across trials for the Kinetic Breaks (*β* = −1.17, SE = 0.48, *t* = −2.41, *p* = .02) and Social Breaks (*β* = −0.94, SE = 0.47, *t* = −2.00, *p* < .05) conditions, as compared to the Control condition (Figure 3b).

Additionally, we conducted a correlation analysis to directly compare individual subject trends in baseline pupil diameter to trends in peak and mean pupil diameter. Baseline pupil diameter significantly correlated with peak pupil diameter (*r* = 0.40, *p* < .001) but not with mean pupil diameter (*r* = 0.17, *p* = .11). The direction of both correlations indicated that subjects with larger overall baseline pupil diameters also had larger overall task-evoked pupil responses (particularly, larger peak pupil responses).

##### Intelligibility

Average intelligibility scores for each for the random assignment groups in Experiment 1 are reported in Table 2. Intelligibility was scored based on the number of keywords correctly repeated back (versus incorrectly repeated back/missed) per sentence. Differences in plurality (e.g., dog vs. dogs) and tense (e.g., cook vs. cooked) were allowed. To confirm that there were no differences in performance across groups, generalized linear mixed-effects regression was used to model the intelligibility data, which was treated as a grouped binomial. Random intercepts for subjects and items were specified. A fixed effect of group did not improve model fit (*χ*^2^(2) = 1.87, *p* = .39), confirming that differences in performance were not present across the random assignment groups.

### 2.4 | Interim discussion

We used the time-course data of the pupil response, as well as summaries of this time-course data into measures of peak and mean pupil diameter per trial, to examine differences among three random assignment conditions. The outcomes of these separate analyses differed. The analysis of the peak pupil response would indicate that participants assigned to both interventions showed greater fatigue of the pupil response than participants assigned to the control group. In contrast, in both the analyses of the mean pupil response and the time-course data (analyzed using growth curve analysis), trends indicated less fatigue of the pupil response for participants assigned to the social breaks condition than the control condition, and more fatigue for participants assigned to the kinetic breaks condition than the control condition.

For this interim discussion, we primarily focus on outcomes from the growth curve analysis of the time-course data because it provides a richer picture of the pupil response than any single summary metric. Based on this data, the social breaks intervention statistically reduced the fatigue of the pupil response across the experiment. However, the amount by which social breaks improved outcomes was substantially less than what we expected based on our pilot data (see Supplemental Materials).

The outcomes of the kinetic breaks condition differed from the social breaks condition. Rather than reducing the fatigue of the pupil response, the kinetic breaks appear to have possibly increased it. However, another factor at play for the kinetic breaks condition is that the magnitude of the pupil response *started* larger than in either of the other two conditions. Similarly, our exploratory analyses of baseline pupil size indicated that the baseline pupil size for participants in the kinetic breaks condition was relatively (but not significantly) larger than in either of the other two conditions. One possible, post hoc explanation for the data is that participants had greater anticipation (or excitement) for playing with the kinetic toys, which were presented to them before they started the task. Although we did not formally assess this possibility, informal feedback from participants was consistent with this interpretation. The overall difference in participants’ state of arousal may explain the differences in baseline pupil size (which indexes tonic activity), which in turn may have impacted the magnitude of the task-evoked pupil response (which indexes phasic activity). Thus, while our kinetic breaks intervention certainly did not reduce the fatigue of the pupil response as desired, this may be due to the effect of the intervention on tonic arousal. Of course, given that we used a between-subjects design, we also cannot rule out differences in the participants randomly assigned to each condition.

Together, the results of Experiment 1 indicate that the type of breaks included in a pupillometry experiment matter. Our evidence indicates that social breaks from the experiment, in which the researcher briefly converses with the subject, may be an effective way to reduce the fatigue of the pupil response. Breaks in which participants do a more exciting activity such as playing with toys, however, appear to increase the subject’s overall state of arousal and the fatigue of the pupil response. If the researcher’s goal is to reduce the change in the task-evoked pupil response across an experiment, social breaks appear to be the more suitable choice.

## 3 | EXPERIMENT 2

In Experiment 2, we aimed to determine whether observation by a researcher (social pressure) during testing would reduce fatigue of the pupil response, and, if so, whether this manipulation combined with the social breaks intervention from Experiment 1 would be especially impactful.

### 3.1 | Method

#### 3.1.1 | Data and code availability statement

Materials, data, and analysis scripts are available from: https://osf.io/jkcx5/. Experiment 2 was not pre-registered, but followed the same analysis plan as Experiment 1.

#### 3.1.2 | Participants

Participants (*n* = 106) were recruited using the Washington University in St. Louis Psychology Human Subjects Pool. Seven participants were excluded due to data loss from blinking, and six participants were excluded due to data loss from poor tracking of the pupil. Our target sample size was 30 per group, but after exclusions, we had 93 participants with valid data (31 control, 32 observation, 30 observation with breaks) due to unintentional over-recruitment. Statistical analyses were not performed until data collection had ended. The age range of the participants was 18–34 years (M = 21.75, SD = 3.28). Participants provided informed consent and received course credit or pay (at a $10/h rate) as compensation for participation in accordance with the Washington University Institutional Review Board. Participants were native speakers of English with self-reported normal hearing. Of the 93 participants included in the sample, 62 self-identified as female, 28 self-identified as male, two as non-binary, and one as agender.

#### 3.1.3 | Materials

The materials were identical to those in Experiment 1.

#### 3.1.4 | Procedure

The same eye-tracking equipment was used for Experiments 1 and 2. However, prior to Experiment 2 our equipment was moved to a different location in the same building. The overall set-up (spacing of equipment and experiment procedures) remained the same, but minor differences in the environment and lighting were unavoidable. Unless explicitly stated, the general procedures were the same for both experiments.

For the observed conditions, the subject completed the task from one side of a two-sided sound-attenuating booth, and the research assistant remained in the opposite side. A window between the two sides of the booth allowed the researcher to observe the subject during the task. Participants were informed that they were being observed, though their back was facing the window during the actual task (to avoid visual distraction in their peripheral view). For the control condition, participants were made explicitly aware that they would not be observed, and that the researcher would be in another room during the task. Lastly, for the observed with breaks condition, social breaks were conducted as in Experiment 1, with the exception that the subject did not leave the testing room. Both the subject and the researcher remained in their respective sides of the sound-attenuating booth and conversed via an intercom system to avoid unnecessary interactions (per COVID-19-related safety procedures). The total duration of the experiment was approximately 45 min for the Control and Observation groups and 55 min for the Observation with Breaks group.

All questionnaires (i.e., demographic information) were completed online for Experiment 2 to limit interaction between the researcher and the participants.

#### 3.1.5 | Data preparation

Pupil data were prepared for analyses following the same process as Experiment 1.

### 3.2 | Results

Analyses followed the same process as Experiment 1. Log-likelihood model comparisons were used to determine whether each effect significantly improved model fit.

#### 3.2.1 | Peak and mean pupil diameter

Random effects for the linear mixed-effects models included intercepts by subject and by item. Fixed effects included group (levels: control, observation, observation with breaks), trial, and the interaction between group and trial.

**FIGURE 5:**
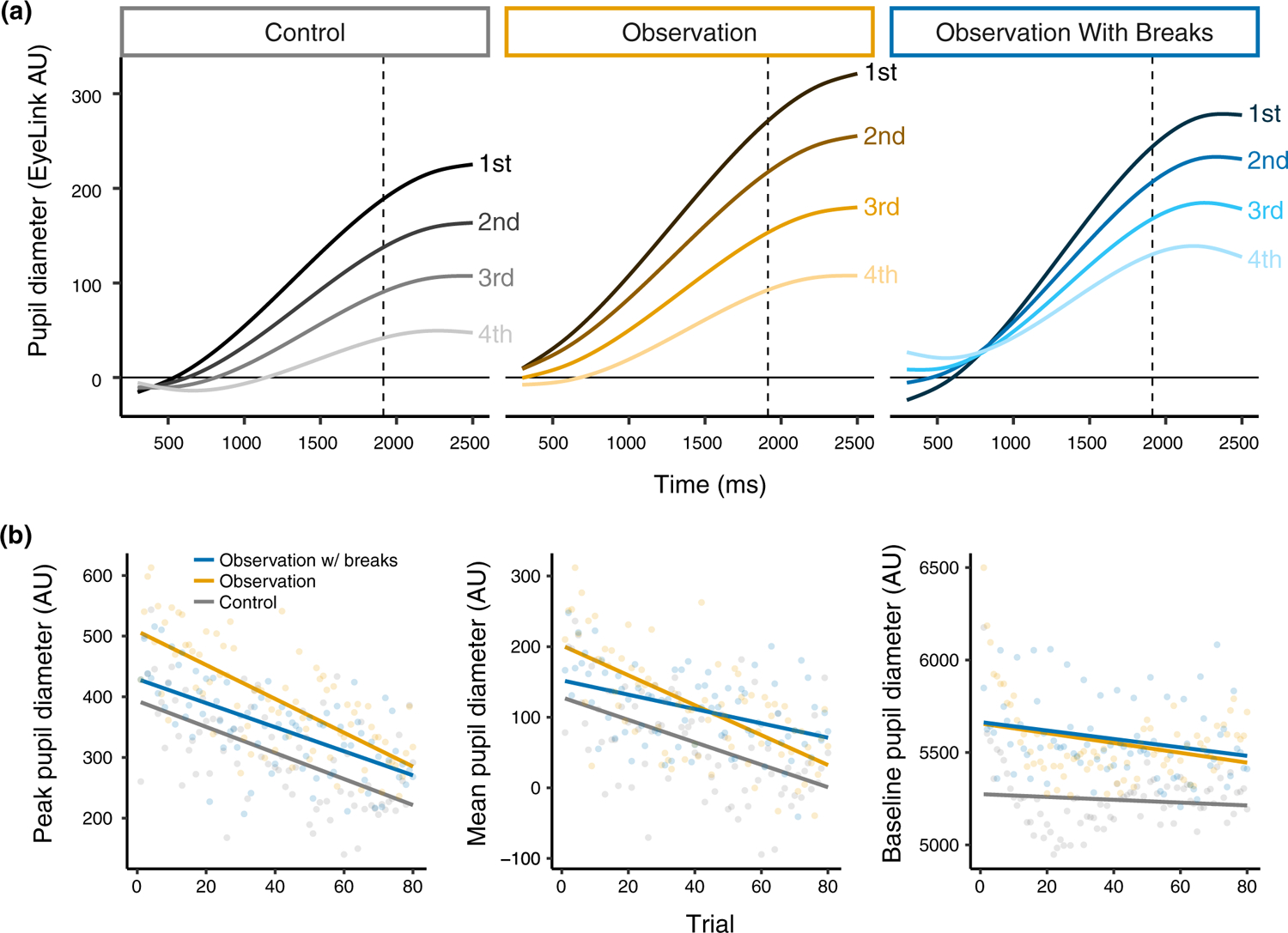
(a) Fatigue of the pupil response in Experiment 2 is visualized by summarizing predicted GCA fits by quartile (i.e., collapsing trials 1–20, 21–40, 41–60, and 61–80). Quartiles of trials are for visualization only and were not used for analyses. Size of the pupil is shown on the y-axis, and time within the trial (where zero is the start of the stimuli) is shown on the x-axis. Dashed vertical lines show the average offset of the stimuli. (b) Changes in peak pupil diameter (left), mean pupil diameter (middle), and baseline pupil diameter (right) across trials are shown for each random assignment group of Experiment 2. Lines represent model fits and points represent mean values of the raw data summarized across all participants for each trial. Note that the peak and mean pupil diameter measures reflect baselined values, whereas baseline pupil diameter is a summary of the raw arbitrary units used for the baselining process.

For peak pupil response, the effect of group improved model fit (*χ*^2^(2) = 7.42, *p* = .02). As shown in Figure 5b, this main effect was primarily driven by the significant difference between the control group and the observation group (*β* = 91.30, SE = 33.41, *t* = 2.73, *p* = .008), with no difference between the control group and the observation with breaks group (*β* = 43.77, SE = 33.95, *t* = 1.29, *p* = .20). The effect of trial was significant (*χ*^2^(1) = 188.12, *p* < .001), and the interaction between trial and group was marginal (*χ*^2^(1) = 4.80, *p* = .09). Across all conditions, the peak pupil response decreased across the course of the task (*β* = −2.26, SE = 0.16, *t* = −13.81, *p* < .001), with some differences in the rate of decrease by group—though none of these differences were significant individually (all *p*’s > .05).

For mean pupil response, the effect of group improved model fit (*χ*^2^(2) = 7.17, *p* = .03). When controlling for the effect of trial, model estimates indicated overall larger mean pupil responses for the observation group than the control group (*β* = 54.50, SE = 21.93, *t* = 2.49, *p* = .01), and overall larger mean pupil responses for the observation with breaks group than the control group (*β* = 47.35, SE = 22.29, *t* = 2.13, *p* = .04). The effect of trial significantly improved model fit (*χ*^2^(1) = 104.48, *p* < .001), and indicated that mean pupil size reduced over the course of the experiment as expected (*β* = −1.57, SE = 0.15, *t* = −10.26, *p* < .001). Lastly, the interaction between group and trial was significant (*χ*^2^(2) = 8.61, *p* = .01). The directions of the trends for the observation and the observation with breaks groups differed: Mean pupil response decreased across the experiment at a somewhat faster rate for the observation group than the control group (*β* = −0.54, SE = 0.37, *t* = −1.46, *p* = .15) and at a somewhat slower rate for the observation with breaks group than the control group (*β* = 0.56, SE = 0.38, *t* = 1.47, *p* = .14; Figure 5b).

#### 3.2.2 | Growth curve analysis

The random effect structure included random intercepts by subject and item, random slopes of the three polynomials by subject, and random slopes of the linear polynomial by item. The random slopes of the quadratic and cubic polynomial within items were initially tested but removed to facilitate model convergence. As in Experiment 1, we were unable to use a full random effect structure, as would be ideal. For fixed effects, we tested the contributions of each polynomial, group, trial, and interactions among each of these factors. Log-likelihood model comparisons were used to determine whether each effect significantly improved model fit (summarized in Table 3), and model summaries were used to examine the direction of effects (summarized in Appendix B).

As in Experiment 1, the linear (*χ*^2^(1) = 87.32, *p* < .001) and cubic (*χ*^2^(1) = 548.32, *p* < .001) polynomials improved model fit, but the quadratic polynomial did not (*χ*^2^(1) = 1.94, *p* = .16). The effect of group did not significantly improve model fit (*χ*^2^(2) = 3.52, *p* = .17), but the effect of trial did (*χ*^2^(1) = 9000.30, *p* < .001).

Model fit was improved significantly by the interaction between group and trial (*χ*^2^(2) = 764.97, *p* < .001). When controlling for all other effects, model estimates indicated that fatigue of the pupil response for the observation group was greater than in the control group (*β* = −0.58, SE = 0.04, *t* = −14.39, *p* < .001). For the observation with breaks group, however, fatigue of the pupil response was reduced over trials as compared to the control group (*β* = 0.54, SE = 0.04, *t* = 13.15, *p* < .001). As shown in Figure 5a, the observation with (social) breaks condition appeared to be the most effective of the three conditions included in Experiment 2. Supplemental Figure S3 shows the non-linear change across trials using raw data (in place of model fits).

The effect of group did not significantly interact with any of the polynomial terms (linear: *χ*^2^(2) = 3.43, *p* = .18; quadratic: *χ*^2^(2) = 0.79, *p* = .67; cubic: *χ*^2^(2) = 3.48, *p* = .18). Interactions between trial and the polynomial terms (linear: *χ*^2^(1) = 3720.10, *p* < .001; quadratic: *χ*^2^(1) = 23.15, *p* < .001; cubic: *χ*^2^(1) = 5.14, *p* = .02) indicated that over the course of the experiment, the pupil response curves became shallower (interaction of linear polynomial and trial: *β* = −10.60, SE = 0.17, *t* = −61.06, *p* < .001) and inflection points became flatter (interaction of quadratic polynomial and trial: *β* = 0.83, SE = 0.17, *t* = 4.81, *p* < .001; interaction of cubic polynomial and trial: *β* = 0.39, SE = 0.17, *t* = 2.27, *p* = .02). These trends in the shape of the pupil response closely matched those from Experiment 1.

#### 3.2.3 | Exploratory analyses of baseline pupil diameter

As in Experiment 1, we conducted exploratory analyses of baseline pupil diameter using linear mixed-effects regression. Figure 5b shows the trends in the data. The effect of trial significantly improved model fit (*χ*^2^(1) = 41.86, *p* < .001), and indicated a decrease in baseline pupil diameter across trials (*β* = −1.75, SE = 0.27, *t* = −6.48, *p* < .001). The effect of group (*χ*^2^(2) = 1.43, *p* = .49) did not significantly improve model fit, and the interaction between trial and group only marginally improved model fit (*χ*^2^(2) = 4.65, *p* < .10). Closer inspection of the interaction of trial and group revealed that baseline pupil size for the observation with breaks group decreased at a significantly faster rate than for the control group (*β* = −1.42, SE = 0.67, *t* = −2.13, *p* = .03), while the observation and control groups did not decrease at significantly different rates (*β* = −0.91, SE = 0.66, *t* = −1.38, *p* = .17).

Lastly, we directly compared individual subject trends in baseline pupil diameter to trends in peak and mean pupil diameter with a correlation analysis. Unlike Experiment 1, baseline pupil diameter did not significantly correlate with either peak pupil diameter (*r* = 0.06, *p* = .56) or mean pupil diameter (*r* = −0.02, *p* = .85).

#### 3.2.4 | Intelligibility

Table 2 reports the average intelligibility scores for each for the random assignment groups in Experiment 2. Intelligibility scoring and modeling matched that for Experiment 1. The overall effect of group did not improve model fit (*χ*^2^(2) = 0.73, *p* = .69), confirming that no significant differences in performance existed between groups.

### 3.3 | Interim discussion

In both the mean pupil response analysis and the growth curve analysis of the time-course data, results indicated more fatigue of the pupil response for the observation group than the control group. For the observation with breaks group, however, results of the time-course data indicated less fatigue of the pupil response than for the control group. When considering all of the data across experiments, it appears that the social breaks intervention was the only one that reduced the fatigue of the pupil response as desired. Both observations of the research participant and interactions with kinetic toys appeared to increase overall tonic arousal (though non-significantly), which may have prevented them from being effective intervention techniques.

## 4 | GENERAL DISCUSSION

In the present study, we aimed to determine the degree to which we could prevent a systematic decrease in the task-evoked pupil response during psychological experiments (“fatigue”). Across two experiments, we found that the fatigue of the task-evoked pupil response was robust. Only social breaks from the experiment, in which the subject conversed briefly with the researcher, reduced fatigue, but the effect was small. For participants given breaks from the experiment during which they interacted with tactile toys and participants who were told that the researcher would be observing them during the task, the fatigue of the pupil response appeared to be more extreme – contrary to our predictions. Regardless of experimental protocol, we observed a dramatic decrease in the magnitude of the pupil response across trials—at least 60% regardless of statistical approach. Below, we consider the possible reasons for this observation and implications for pupillometry research.

Because of well-known interactions between tonic and phasic activity in the locus coeruleus (Aston-Jones & Cohen, 2005; Berridge & Waterhouse, 2003), we predicted that the fatigue of the task-evoked pupil response would likely be linked to a change in tonic activity. We examined the baseline pupil size directly preceding each trial, expecting that as participants became less engaged with the task (due to fatigue, habituation, or boredom), baseline pupil diameter would decrease, and the amplitude of the task-evoked pupil response along with it (as found by Pielage et al., 2021; c.f., Hopstaken et al., 2015). Our results indeed indicated a steady decrease in baseline pupil diameter across conditions; however, this change was actually least dramatic in the control conditions of each experiment. Our data suggest that some types of breaks (such as those with tactile toys) may actually elevate the overall, initial baseline pupil diameter, resulting in more change in pupil response amplitude across the experiment. Further, the decrease in baseline pupil diameter across the task was only (modestly) correlated with peak pupil diameter in Experiment 1, and not Experiment 2. Unfortunately, these conflicting results do not clarify the relationship between the fatigue of the task-evoked pupil response and baseline pupil diameter, for which there was already mixed data (Pielage et al., 2021; c.f., Hopstaken et al., 2015).

There are several important methodological implications of our current study. The first is that it is difficult, if not impossible, to remove fatigue effects from pupillometry studies by providing breaks with or without the opportunity for social or other types of interactions (e.g., interaction with kinetic toys). Even the most promising of our manipulations—breaks with a social aspect—had a relatively small effect on these fatigue effects. Thus, in the absence of evidence, pupillometry researchers cannot assume that any particular experimental protocol obviates the need to consider fatigue effects.

Given that fatigue effects seem here to stay, what does that mean for pupillometry research? One important consequence is that trial types that are statistically compared should be close together in time. That is, because the pupil response systematically varies as a function of time, if experimental conditions also vary as a function of time—for example, if condition A is always first, followed by condition B—the task-evoked pupil response will differ between the two tasks as a function of fatigue effects. This consideration may be particularly important for studies focused on learning or habituation, in which disentangling learning effects from fatigue effects is likely to be particularly challenging.

Another implication of our findings is that modeling the change in pupil response across trials is critically important. Including time as a predictor dramatically improves model fit, and accounts for a large source of variance in the data. The nature of these time-based predictors (e.g., linear vs. quadratic) may depend on the specific task or design, but they can easily be explored and tested.

Although the present paper does not include power analyses of each analysis type, our results suggest that modeling the time course of the data with growth curve analysis may have provided more power than modeling summary statistics (i.e., peak and mean pupil diameters). A direct methodological inquiry examining other cognitive pupillometry experiments is necessary to verify whether this may be the case. While this observation is not specific to fatigue effects, it does suggest growth curve analyses provide an elegant way to account for changes in the pupil response over time (see also Hershman et al., 2022).

One limitation of the present work is that the effectiveness of each intervention was only tested with one type of psychological experiment, and with one set amount of breaks. Given our research group’s interests, we opted to examine fatigue effects for a speech-in-noise perception task. Examining these and similar interventions with different kinds of psychological paradigms (e.g., *n*-back tasks; Hopstaken et al., 2015), and with different numbers of breaks, remains an open avenue for future research. Further, our examination did not compare fatigue effects for tasks ranging in difficulty. Indeed, because the construct of fatigue is complex (involving both subjective feelings well as physical decrements; Tiesinga et al., 1996; Chaudhuri & Behan, 2000; Hornsby et al., 2016), it is possible that more highly demanding pupillometry experiments may be better served by different interventions.

Finally, it is important to note that we did not design our study to evaluate other possible effects of interventions. For example, on some tasks, frequent social interactions with researchers may improve behavioral performance or interact with constructs of interest such as sustained attention—even if the effect on the pupil response is minimal. In the current study, differences in intelligibility (performance) did not emerge between the random assignment conditions. However, average intelligibility across all conditions and both experiments was high (approximately 90%), which could indicate a ceiling effect. It is possible that, in a more challenging task, these interventions or others would be able to better differentiate performance levels or prove beneficial by other metrics.

## 5 | CONCLUSIONS

Fatigue effects are a ubiquitous concern in pupillometry research. Our current results suggest the following conclusions:

1. Fatigue of the pupil response is difficult or impossible to avoid;
2. Although social breaks may help to a degree, they do not entirely prevent fatigue of the pupil response;
3. Given the presence of fatigue effects, researchers need to account for fatigue in both experimental design and statistical analysis (e.g., modeling time across an experiment).
4. By better understanding how the pupil response changes over the course of an experiment we can make even better use of this promising tool.

## Supplementary Material

supplementary material

## Figures and Tables

**TABLE 1:** Log-likelihood model comparisons for the growth curve analysis of Experiment 1

Effect	*χ* ^*2*^	*df*	*p*
Linear polynomial	59.16	1	<.001
Quadratic polynomial	0.01	1	.91
Cubic polynomial	30.88	1	<.001
Group	1.15	2	.56
Trial	11,705	1	<.001
Group × Trial	158.90	2	<.001
Group × Linear polynomial	1.20	2	.55
Group × Quadratic polynomial	2.91	2	.23
Group × Cubic polynomial	1.75	2	.42
Trial × Linear polynomial	3525.60	1	<.001
Trial × Quadratic polynomial	58.38	1	<.001
Trial × Cubic polynomial	19.85	1	<.001

**TABLE 2:** Intelligibility summaries for Experiments 1 and 2

Experiment 1		Experiment 2	
Condition	Proportion correct	Condition	Proportion correct
Control	0.90 (0.18)	Control	0.90 (0.17)
Kinetic Breaks	0.90 (0.18)	Observation	0.91 (0.17)
Social Breaks	0.91 (0.17)	Observation with Breaks	0.91 (0.17)

*Note*: Means are reported with standard deviations in parentheses.

**TABLE 3:** Log-likelihood model comparisons for the growth curve analysis of Experiment 2

Effect	*χ* ^*2*^	*df*	*p*
Linear polynomial	87.32	1	<.001
Quadratic polynomial	1.94	1	.16
Cubic polynomial	548.32	1	<.001
Group	3.52	2	.17
Trial	9000.30	1	<.001
Group × Trial	764.97	2	<.001
Group × Linear polynomial	3.43	2	.18
Group × Quadratic polynomial	0.79	2	.67
Group × Cubic polynomial	3.48	2	.18
Trial × Linear polynomial	3720.10	1	<.001
Trial × Quadratic polynomial	23.15	1	<.001
Trial × Cubic polynomial	5.14	1	.02

## Data Availability

The data that support the findings of this study are openly available in OSF at https://osf.io/jkcx5/files/osfstorage.

## APPENDIX A Growth Curve Analysis Models from Experiment 1

**TABLE A1:** Model with all lower-order fixed effects order fixed

Fixed effects				
Predictor	Coefficient (*β*)	S.E.	*t*	*p*
Intercept	87.31	8.05	10.85	<.001
Linear polynomial	417.10	46.62	8.95	<.001
Quadratic polynomial	1.74	15.04	0.12	.91
Cubic polynomial	−59.20	10.10	−5.86	<.001
Group (Kinetic Breaks)	5.05	5.64	0.90	.37
Group (Social Breaks)	5.42	5.54	0.98	.33
Trial	−1.16	0.01	−108.61	<.001

Random effects					
	Variance	S.D.	Correlation Matrix		
Subject (Intercept)	4119.00	64.18			
Subject (Linear polynomial slope)	166534.80	408.09	0.89		
Subject (Quadratic polynomial slope)	12955.00	113.82	−0.23	0.08	
Subject (Cubic polynomial slope)	48.35.90	69.54	−0.59	−0.69	0.25
Item (Intercept)	545.20	23.35			
Item (Linear polynomial slope)	20196.20	142.11			
Item (Quadratic polynomial slope)	5645.20	75.13			
Item (Cubic polynomial slope)	3184.00	56.34			

**TABLE A2:** Model with all lower-effects and interactions

Fixed effects				
Predictor	Coefficient (*β*)	S.E.	*t*	*p*
Intercept	87.58	12.22	7.17	<.001
Linear polynomial	660.50	77.08	8.57	<.001
Quadratic polynomial	−54.05	23.30	−2.32	.02
Cubic polynomial	−81.84	15.60	−5.25	<.001
Group (Kinetic Breaks)	15.03	17.03	0.88	.38
Group (Social Breaks)	−4.51	16.74	−0.27	.79
Trial	−1.17	0.02	−63.53	<.001
Group (Kinetic Breaks): Trial	−0.15	0.03	−5.79	<.001
Group (Social Breaks): Trial	0.18	0.03	6.81	<.001
Linear polynomial: Group (Kinetic Breaks)	94.02	107.40	0.88	.38
Linear polynomial: Group (Social Breaks)	−14.84	105.60	−0.14	.89
Quadratic polynomial: Group (Kinetic Breaks)	50.72	30.34	1.67	.10
Quadratic polynomial: Group (Social Breaks)	14.42	29.82	0.48	.63
Cubic polynomial: Group (Kinetic Breaks)	−9.19	19.31	−0.48	.64
Cubic polynomial: Group (Social Breaks)	15.90	18.97	0.84	.40
Linear polynomial: Trial	−6.66	0.11	−59.45	<.001
Quadratic polynomial: Trial	0.86	0.11	7.64	<.001
Cubic polynomial: Trial	0.50	0.11	4.46	<.001

Random effects					
	Variance	S.D.	Correlation Matrix		
Subject (Intercept)	4111.70	64.12			
Subject (Linear polynomial slope)	163786.70	404.71	0.89		
Subject (Quadratic polynomial slope)	12539.20	111.98	−0.24	0.06	
Subject (Cubic polynomial slope)	4735.20	68.81	−0.59	0.69	0.27
Item (Intercept)	543.50	23.31			
Item (Linear polynomial slope)	20241.50	142.27			
Item (Quadratic polynomial slope)	5640.30	75.10			
Item (Cubic polynomial slope)	3196.90	56.54			

*Note*: Summaries of the linear mixed-effects regression models for the growth curve analysis. Table A1 shows the model containing all lower-order fixed effects, and Table A2 shows the model containing both lower-order effects and two-way interactions. Reference level in dummy-coding for Group is Control.

Abbreviations: *S.D*., Standard Deviation; *S.E*., Standard Error.

## APPENDIX B Growth Curve Analysis Models from Experiment 2

**TABLE B1:** Model with all lower-order fixed effects

Fixed effects				
Predictor	Coefficient (*β*)	S.E.	*t*	*p*
Intercept	150.40	11.48	13.11	<.001
Linear polynomial	709.10	61.58	11.51	<.001
Quadratic polynomial	−27.76	19.83	−1.40	.16
Cubic polynomial	−93.36	12.49	−7.47	<.001
Group (Kinetic Breaks)	15.99	9.75	1.64	.10
Group (Social Breaks)	16.98	9.91	1.71	.09
Trial	−1.57	0.02	−95.14	<.001

Random effects					
	Variance	S.D.	Correlation Matrix		
Subject (Intercept)	7623	87.31			
Subject (Linear polynomial slope)	300,618	548.29	0.75		
Subject (Quadratic polynomial slope)	35,086	187.31	−0.46	−0.11	
Subject (Cubic polynomial slope)	13,042	114.20	−0.33	−0.71	0.19
Item (Intercept)	1357	36.83			
Item (Linear polynomial slope)	43,516	208.60	0.59		

**TABLE B2:** Model with all lower-order fixed effects and interactions

Fixed effects					
Predictor	Coefficient (*β*)		S.E.	*t*	*p*
Intercept	126.00		15.98	7.88	<.001
Linear polynomial	986.90		99.59	9.91	<.001
Quadratic polynomial	−37.24		34.87	−1.07	.29
Cubic polynomial	−92.79		22.34	−4.15	<.001
Group (Kinetic Breaks)	78.10		21.67	3.61	<.001
Group (Social Breaks)	26.34		22.02	1.20	.23
Trial	−1.54		0.03	−53.79	<.001
Group (Kinetic Breaks): Trial	−0.58		0.04	−14.39	<.001
Group (Social Breaks): Trial	0.54		0.04	13.15	<.001
Linear polynomial: Group (Kinetic Breaks)	206.40		135.40	1.52	.13
Linear polynomial: Group (Social Breaks)	233.60		137.60	1.70	.09
Quadratic polynomial: Group (Kinetic Breaks)	−29.88		47.95	−0.62	.53
Quadratic polynomial: Group (Social Breaks)	−42.12		48.72	−0.86	.39
Cubic polynomial: Group (Kinetic Breaks)	−0.33		29.78	−0.01	.99
Cubic polynomial: Group (Social Breaks)	−49.55		30.27	−1.64	.11
Linear polynomial: Trial	−10.60		0.17	−61.06	<.001
Quadratic polynomial: Trial	0.83		0.17	4.81	<.001
Cubic polynomial: Trial	0.39		0.17	2.27	0.02

Random effects					
	Variance	S.D.	Correlation Matrix		
Subject (Intercept)	7338	85.66			
Subject (Linear polynomial slope)	287,387	536.08	0.74		
Subject (Quadratic polynomial slope)	34,724	186.34	−0.45	−0.09	
Subject (Cubic polynomial slope)	12,502	111.81	−0.33	−0.72	0.18
Item (Intercept)	1362	36.90			
Item (Linear polynomial slope)	22,097	209.99	0.60		

*Note*: Summaries of the linear mixed-effects regression models for the growth curve analysis. Table B1 shows the model containing all lower-order fixed effects, and Table B2 shows the model containing both lower-order effects and two-way interactions. Reference level in dummy-coding for Group is Control.

Abbreviations: *S.D*., Standard Deviation; *S.E*., Standard Error.

## References

[R1] Aston-Jones G, & Cohen JD (2005). An integrative theory of locus coeruleus-norepinephrine function: Adaptive gain and optimal performance. Annual Review of Neuroscience, 28, 403–450. 10.1146/annurev.neuro.28.061604.135709 16022602

[R2] Beatty J (1982). Task-evoked pupillary responses, processing load, and the structure of processing resources. Psychological Bulletin, 91(2), 276–292. 10.1037/0033-2909.91.2.276 7071262

[R3] Berridge CW, & Waterhouse BD (2003). The locus coeruleus–noradrenergic system: Modulation of behavioral state and state-dependent cognitive processes. Brain Research Reviews, 42(1), 33–84. 10.1016/S0165-0173(03)00143-7 12668290

[R4] Boersma P, & Weenink D (2019). Doing phonetics by computer (version 6.0. 46).

[R5] Brown VA, McLaughlin DJ, Strand JF, & Van Engen K (2020). Rapid adaptation to fully intelligible nonnative-accented speech reduces listening effort. Quarterly Journal of Experimental Psychology, 73, 1431–1443. 10.1177/1747021820916726 32192390

[R6] Chaudhuri A, & Behan PO (2000). Fatigue and basal ganglia. Journal of the Neurological Sciences, 179(1–2), 34–42. 10.1016/S0022-510X(00)00411-1 11054483

[R7] Geller J, Winn MB, Mahr T, & Mirman D (2020). GazeR: A package for processing gaze position and pupil size data. Behavior Research Methods, 52, 2232–2255. 10.3758/s13428-020-01374-8 32291732 PMC7544668

[R8] Gilzenrat MS, Cohen JD, Rajkowski J, & Aston-Jones G (2003). Pupil dynamics predict changes in task engagement mediated by locus coeruleus. Society for Neuroscience Abstracts, 515, 19.

[R9] Hershman R, Milshtein D, & Henik A (2022). The contribution of temporal analysis of pupillometry measurements to cognitive research. Psychological Research, 1–15. 10.1007/s00426-022-01656-0 35178621

[R10] Hopstaken JF, Van Der Linden D, Bakker AB, & Kompier MA (2015). The window of my eyes: Task disengagement and mental fatigue covary with pupil dynamics. Biological Psychology, 110, 100–106. 10.1016/j.biopsycho.2015.06.013 26196899

[R11] Hornsby BW, Naylor G, & Bess FH (2016). A taxonomy of fatigue concepts and their relation to hearing loss. Ear and Hearing, 37(Suppl 1), 136S. 10.1097/AUD.0000000000000289 27355763 PMC4930001

[R12] Jiang Y, Snedeker J, & Rabagliati H (2017). Growth curves and autocorrelations. RStudio Pubs. http://rstudio-pubs-static.s3.am-azonaws.com/267836_461919185abd4053aa56b3209363d3ab.html

[R13] Kahneman D, & Beatty J (1966). Pupil diameter and load on memory. Science, 154, 1583–1585. 10.1126/science.154.3756.1583 5924930

[R14] Laeng B, Sirois S, & Gredebäck G (2012). Pupillometry: A window to the preconscious? Perspectives on Psychological Science: A Journal of the Association for Psychological Science, 7, 18–27. 10.1177/1745691611427305 26168419

[R15] McGarrigle R, Dawes P, Stewart AJ, Kuchinsky SE, & Munro KJ (2017). Pupillometry reveals changes in physiological arousal during a sustained listening task. Psychophysiology, 54(2), 193–203. 10.1111/psyp.12772 27731503

[R16] McLaughlin DJ, & Van Engen KJ (2020). Task-evoked pupil response for accurately recognized accented speech. The Journal of the Acoustical Society of America, 147(2), EL151–EL156. 10.1121/10.0000718 32113314

[R17] Mirman D (2017). Growth curve analysis and visualization using R. Chapman and Hall/CRC. 10.1201/9781315373218

[R18] Morad Y, Lemberg H, Yofe N, & Dagan Y (2000). Pupillography as an objective indicator of fatigue. Current Eye Research, 21(1), 535–542. 10.1076/0271-3683(200007)2111-ZFT535 11035533

[R19] Nichols AL, & Maner JK (2008). The good-subject effect: Investigating participant demand characteristics. The Journal of General Psychology, 135(2), 151–165. 10.3200/GENP.135.2.151-166 18507315

[R20] Peelle JE, & Van Engen KJ (2021). Time stand still: Effects of temporal window selection on eye tracking analysis. Collabra: Psychology, 7. 10.1525/collabra.25961 PMC8752058 35024541

[R21] Pielage H, Zekveld AA, Saunders GH, Versfeld NJ, Lunner T, & Kramer SE (2021). The presence of another individual influences listening effort, but not performance. Ear and Hearing, 42(6), 1577–1589. 10.1097/AUD.0000000000001046 33795615 PMC8542087

[R22] Plain B, Pielage H, Richter M, Bhuiyan TA, Lunner T, Kramer SE, & Zekveld AA (2021). Social observation increases the cardiovascular response of hearing-impaired listeners during a speech reception task. Hearing Research, 410, 108334. 10.1016/j.heares.2021.108334 34450568

[R23] Raftery AE (1995). Bayesian model selection in social research. Sociological methodology, 111–163. 10.2307/271063

[R24] Rajkowski J (1993). Correlations between locus coeruleus (LC) neural activity, pupil diameter and behavior in monkey support a role of LC in attention. Society for Neuroscience. https://ci.nii.ac.jp/naid/10021384962/

[R25] Reilly J, Kelly A, Kim SH, Jett S, & Zuckerman B (2019). The human task-evoked pupillary response function is linear: Implications for baseline response scaling in pupillometry. Behavior Research Methods, 51(2), 865–878. 10.3758/s13428-018-1134-4 30264368

[R26] Samuels ER, & Szabadi E (2008). Functional neuroanatomy of the noradrenergic locus coeruleus: Its roles in the regulation of arousal and autonomic function part I: Principles of functional organisation. Current Neuropharmacology, 6(3), 235–253. 10.2174/157015908785777229 19506723 PMC2687936

[R27] R Core Team. (2013). R: A language and environment for statistical computing. R Foundation for Statistical Computing. http://www.R-project.org/

[R28] Tiesinga LJ, Dassen TW, & Halfens RJ (1996). Fatigue: A summary of the definitions, dimensions, and indicators. International Journal of Nursing Terminologies and Classifications, 7(2), 51–62. 10.1111/j.1744-618X.1996.tb00293.x 8716946

[R29] Tryon WW (1975). Pupillometry: A survey of sources of variation. Psychophysiology, 12(1), 90–93. 10.1111/j.1469-8986.1975.tb03068.x 1114216

[R30] Van Engen KJ, Chandrasekaran B, & Smiljanic R (2012). Effects of speech clarity on recognition memory for spoken sentences. PLoS One, 7(9), e43753. 10.1371/journal.pone.0043753 22970141 PMC3436755

[R31] Van Engen KJ, & McLaughlin DJ (2018). Eyes and ears: Using eye tracking and pupillometry to understand challenges to speech recognition. Hearing Research, 369, 56–66. 10.1016/j.heares.2018.04.013 29801981 PMC7101020

[R32] van Rij J, Hendriks P, van Rijn H, Baayen RH, & Wood SN (2019). Analyzing the time course of pupillometric data. Trends in Hearing, 23, 2331216519832483. 10.1177/2331216519832483 PMC6535748 31081486

[R33] Wang CA, Baird T, Huang J, Coutinho JD, Brien DC, & Munoz DP (2018). Arousal effects on pupil size, heart rate, and skin conductance in an emotional face task. Frontiers in Neurology, 9, 1029. 10.3389/fneur.2018.01029 30559707 PMC6287044

[R34] Wickham H, François R, Henry L, & Müller K (2021). dplyr: A grammar of data manipulation. R package version 1.0.7. https://CRAN.R-project.org/package=dplyr

[R35] Winn MB, Wendt D, Koelewijn T, & Kuchinsky SE (2018). Best practices and advice for using pupillometry to measure listening effort: An introduction for those who want to get started. Trends in Hearing, 22, 2331216518800869. 10.1177/2331216518800869 PMC6166306 30261825

