# Supplementary material for: Give me a break! Unavoidable fatigue effects in cognitive pupillometry
Source: Psychophysiology. Author manuscript; Available in PMC 2024 Jun 8. (PMC11161670; doi:10.1111/psyp.14256)
Supplement: supplementary material [file NIHMS1998931-supplement-supplementary_material.pdf]

Supplemental Materials for "Give me a break! Unavoidable fatigue effects in cognitive pupillometry"

Drew J. McLaughlin[1], Maggie Zink[2], Lauren Gaunt[1], Jamie Reilly[3],
Mitchell S. Sommers[1], Kristin J. Van Engen[1], Jonathan E. Peelle[2]

[1] Department of Psychological and Brain Sciences, Washington University in Saint Louis
[2] Department of Otolaryngology, Washington University in Saint Louis
[3] Department of Communication Sciences and Disorders, Temple University

Pilot data

Methods. *Stimuli.* Recordings of two Mandarin Chinese-accented (nonnative) speakers of English reading semantically normal sentences (the same sentences used in Experiments 1 and 2; Van Engen et al., 2012) were used in the pilot task. Sentences were presented in speech-shaped noise at 0 dB SNR. Two speakers were included in the stimuli set in order to determine their relative intelligibilities (as determined by pupil response) as part of an aim for a different study. For the purposes of the current study, we ignore this within-subject manipulation and report only the change in pupil response over the course of the experiment as a function of the between-participants manipulation.

Procedure. All participants provided written informed consent prior to participation. Set up of all equipment matched that in Experiment 1. Of the 12 participants in the pilot, six were assigned to the control condition (with no breaks or observation) and the other six were assigned to the intervention condition (with breaks and observation; see *observation with breaks* condition in Experiment 2).

The task began with a practice section lasting six trials (three sentences per speaker). Next, participants completed 80 test trials (40 per speaker), with breaks interspersed every five trials for the Intervention group only. During breaks, participants chatted with the research assistant briefly (similar to the protocol for the Social Breaks condition in Experiment 1). Breaks

were untimed for the pilot, and did not require the subject to leave the sound-attenuating booth. Calibration of the EyeLink camera to the pupil was visually inspected by the research assistant before the participant resumed the experiment after each break.

The trial flow and procedures of the pilot task matched that of Experiments 1 and 2, with the exception of the inter-stimulus interval, which was not included. The 80 audio files were presented in a random order.

Results. Growth curve analysis was used to examine the data from the pilot task. Steps for selecting a window of analysis matched those in Experiments 1 and 2. An analysis window between 1500 ms and 5800 ms was selected for this dataset. Random effects in the models included random intercepts by participants and items, and random slopes of the linear, quadratic, and cubic polynomials by subject. Correlations among the random slopes were removed to avoid overfitting.

Fixed effects included the linear, quadratic, and cubic orthogonal polynomials, group, trial, the interaction between group and trial, the interactions between each polynomial and group, and the interactions between each polynomial and trial. A summary of the log-likelihood model comparisons is presented in **Supplemental Table 1**.

Supplemental Table 1. Log-likelihood model comparisons for the growth curve analysis of the pilot data

**EFFECT**	$\chi^2(1)$	***p***
Linear polynomial	14.23	< .001
Quadratic polynomial	0.65	.42
Cubic polynomial	14.13	< .001
Group	0.70	.40
Trial	2881.10	< .001
Group x Trial	600.95	< .001
Linear polynomial x Group	0.53	.47
Quadratic polynomial x Group	0.10	.75
Cubic polynomial x Group	0.77	.38
Linear polynomial x Trial	903.29	< .001
Quadratic polynomial x Trial	14.91	< .001
Cubic polynomial x Trial	17.07	< .001

Most notably, the significant interaction between group and trial ($\chi^2(1) = 600.95$, $p < .001$) robustly indicated less fatigue of the pupil response for participants in the intervention condition than the control condition ($\beta = 2.93$, SE = 0.12, $t = 24.53$, $p < .001$). **Supplemental Figure 1** shows the model fits as well as smooth lines of the raw data fits, summarized by quartiles of the experiment for visualization purposes.

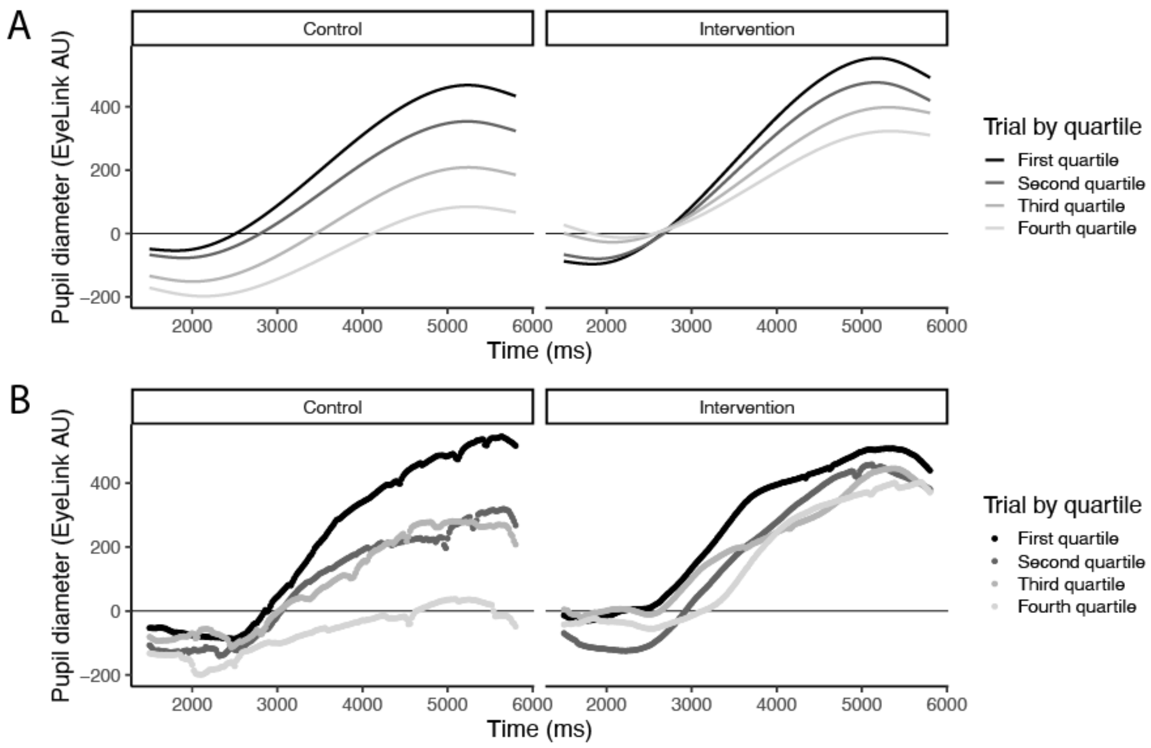

Supplemental Figure 1. A) Fatigue of the pupil response in the pilot experiment is visualized by summarizing predicted fit data by quartile (i.e., collapsing trials 1-20, 21-40, 41-60, and 61-80). Quartiles of trials are for visualization only and were not used for analyses. Size of the pupil is shown on the y-axis, and time within the trial is shown on the x-axis. **B)** This same dataset is visualized with raw data points in place of model fits to show non-linearity in change across trials.

Non-linear change across trials

In this supplemental section we present raw data trends from Experiments 1 and 2. Although change across trials was modeled linearly in our primary analyses, we note that the fatigue of the pupil response does not necessarily decrease in equal intervals across the experiment. In Experiment 1 (**Supplemental Figure 2**), a larger amount of change is seen between the first quartile (trials 1-20) and the second quartile (trials 21-40) of the experiment than between the other quartiles. In Experiment 2 (**Supplemental Figure 3**), this also appears to be the case, although to a lesser degree. It may be that nonlinear modeling of fatigue responses will improve model accuracy in future work.

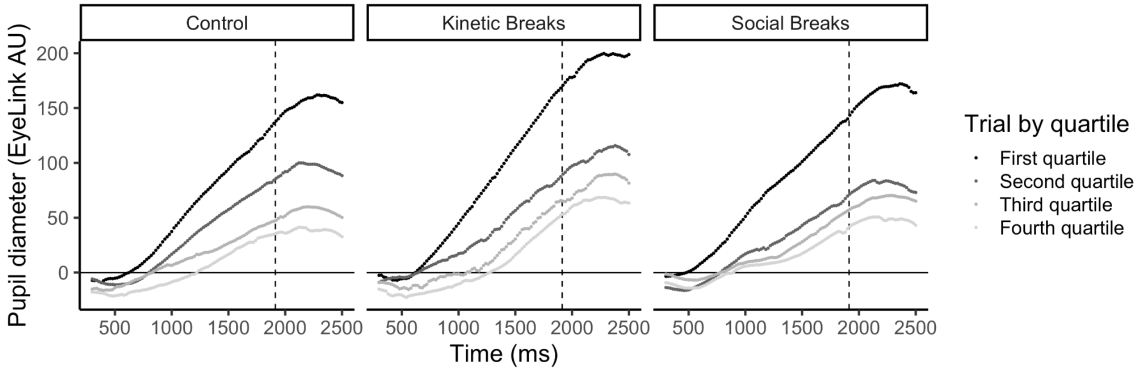

Supplemental Figure 2. Fatigue of the pupil response in Experiment 1 is visualized by summarizing raw data means by quartile (i.e., collapsing trials 1-20, 21-40, 41-60, and 61-80). Quartiles of trials are for visualization only and were not used for analyses. Size of the pupil is shown on the y-axis, and time within the trial (where zero is the start of the stimuli) is shown on the x-axis. Dashed vertical lines show the average offset of the stimuli.

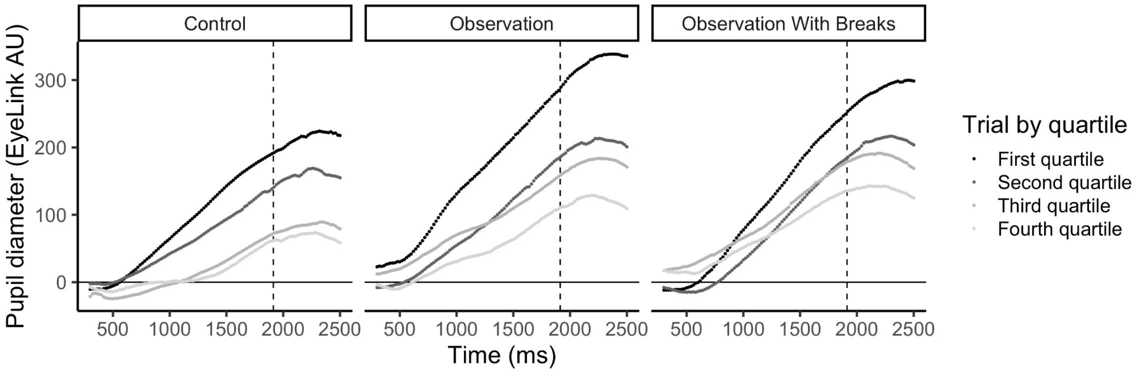

Supplemental Figure 3. Fatigue of the pupil response in Experiment 2 is visualized by summarizing raw data means by quartile (i.e., collapsing trials 1-20, 21-40, 41-60, and 61-80). Quartiles of trials are for visualization only and were not used for analyses. Size of the pupil is shown on the y-axis, and time within the trial (where zero is the start of the stimuli) is shown on the x-axis. Dashed vertical lines show the average offset of the stimuli.
